# Editorial: Triple-negative breast cancer: Heterogeneity, tumor microenvironment and targeted therapy

**DOI:** 10.3389/fonc.2022.1026566

**Published:** 2022-11-21

**Authors:** Xiyun Deng, Chanjuan Zheng, Faqing Tang, Thomas J. Rosol, Zhi-Ming Shao

**Affiliations:** ^1^ Key Laboratory of Translational Cancer Stem Cell Research, Hunan Normal University School of Medicine, Changsha, Hunan, China; ^2^ Clinical Laboratory of Hunan Cancer Hospital and the Affiliated Cancer Hospital of Xiangya School of Medicine, Central South University, Hunan Key Laboratory of Oncotarget Gene, Changsha, Hunan, China; ^3^ Department of Biomedical Sciences, Heritage College of Osteopathic Medicine, Ohio University, Athens, OH, United States; ^4^ Key Laboratory of Breast Cancer in Shanghai, Department of Breast Surgery, Fudan University Shanghai Cancer Center, Shanghai Medical College, Fudan University, Shanghai, China; ^5^ Department of Oncology, Shanghai Medical College, Fudan University, Shanghai, China

**Keywords:** triple-negative breast cancer, heterogeneity, tumor microenvironment, targeted therapy, clinical outcomes

## Introduction

Triple-negative breast cancer (TNBC), a complex subtype of breast cancer that lacks estrogen receptor (ER), progesterone receptor (PR), and human epidermal growth factor receptor 2 (HER2), is characterized by aggressive behavior, high incidence of relapse, and unfavorable prognosis (1). Emerging targeted therapeutic strategies currently approved for the clinical treatment of TNBC include poly (ADP-ribose) polymerase (PARP) inhibitors (2), immune checkpoint inhibitors (ICIs) (3), and antibody-drug conjugates (ADCs) (4). Although some improvements have been observed in survival outcomes, the overall efficacy in unselected TNBC patients remains unsatisfactory. It is reported that the response rate of ICI monotherapy in TNBC ranges between 5% and 25% (5). The reasons for treatment refraction are many and are at least partly attributable to the heterogeneity of the tumor microenvironment (6, 7). Novel therapeutic options for different subtypes of TNBC, particularly those taking into consideration the unique biological features and the highly heterogeneous nature of the tumor microenvironment in different subtypes, are urgently needed and have become an area of active investigation in TNBC research.

In this Research Topic, we present the theme “*TNBC: Heterogeneity, Tumor Microenvironment and Targeted Therapy*” through 11 articles including 5 original research papers, 5 review (or mini-review) articles and 1 bibliometric analysis. The original research papers include one that focuses on TNBC tumor microenvironment (Wang et al.), one that describes about a nomogram model for predicting distant metastasis of lymph node-negative TNBC (Peng et al.), one that presents potential novel therapeutic strategy targeting intracellular signaling pathway in TNBC (Cui et al.), and one that identifies cancer stem cells as a novel cellular target for TNBC (Zheng et al.). A bibliometric analysis of the research hotspots in TNBC is also included (Hao et al.). The 5 review (or mini-review) articles cover tumor subtyping (Ensenyat-Mendez et al.) and targeted therapies particularly focusing on targeting the tumor immune microenvironment (Li et al., Yi et al., Tan et al. and Clark and Yang).

## Novel strategies of targeted therapies for TNBC

As mentioned above, although targeted therapeutic strategies have achieved clinical benefit in some patients, the overall responses in unselected TNBC patients are still limited. Therefore, there is an urgent need in developing more robust targeted approaches for improving the outcomes in TNBC. Signaling pathways that are under active investigation as potential targets for TNBC include intracellular signaling such as tyrosine kinases, as well as cell cycle regulation, DNA damage and cell death regulation, etc (8, 9). Protein tyrosine kinases (PTKs) are a group of enzymes that can transfer a phosphate group from ATP to the tyrosine residues of specific proteins inside a cell. Phosphorylation of proteins by PTKs is an important mechanism of intracellular signaling that regulates diverse cellular processes, e.g., cell division. Classic PTK inhibitors, such as imatinib and osimertinib, that have achieved excellent efficacy in other cancers have failed to meet the same expectations in TNBC. Cui et al. analyzed breast cancer tissues for the expression of PTK7, a member of the PTK superfamily, which plays a critical role in tumor development and progression. They found that high expression of PTK7 significantly correlated with high rates of metastasis and poor prognosis in TNBC patients (Cui et al.). Whether these novel signaling molecules can be explored as therapeutic targets for TNBC needs to be further evaluated.

Statins are well known for their lipid-lowering effects in patients with cardiovascular disease. The recently recognized anti-cancer activity of statins may be due to their pleiotropic effects, including targeting cancer stem cells, a small heterogeneous population of cancer cells that contributes to tumor initiation, metastasis, and recurrence. Through LC-MS/MS-based proteomics and lysine acylation profiling, researchers at Hunan Normal University demonstrated that lovastatin, a naturally occurring lipophilic statin, inhibits TNBC cancer stem cells by dysregulating the cytoskeleton, thus suppressing epithelial-to-mesenchymal transition (EMT) and metastasis (Zheng et al.). Other old drugs that have been shown to inhibit TNBC cancer stem cells include mifepristone (10), metformin (11), disulfiram (12), salinomycin (13), etc. Mifepristone and metformin have been examined in phase I clinical trials in solid tumors including TNBC (NCT02014337 for mifepristone and NCT01650506 for metformin). Although these trials have been completed 5 years ago, no results have been posted yet. Disulfiram has been evaluated examined in two phase II trials in metastatic breast cancer (NCT03323346, NCT04265274), pending release of trial results. These drugs should be examined in better designed clinical studies for their potential for repositioning as clinically beneficial drugs.

## Biomarkers to predict the risk and therapeutic efficacy of TNBC

Prediction models can be an excellent tool to identify the patients at high risk. The poor prognosis of lymph node-negative TNBC has been well documented, but reliable biomarkers to predict those at increased risk of metastasis are still lacking. Researchers at Fudan University Shanghai Cancer Center generated a nomogram by incorporating a seven-gene signature with clinical parameters, including patient age and tumor size. This composite model shows improved prognostic accuracy and holds promise for individualized treatment by identifying lymph node-negative TNBC patients who are at a higher risk of distant metastasis (Peng et al.).

Clinical trials have demonstrated that PDL1-positive advanced TNBC patients benefit from atezolizumab-based ICI plus chemotherapy. Biomarkers for PD1/PDL1-targeted ICI therapy include PDL1 expression level, tumor mutational burden (TMB), and tumor-infiltrating lymphocytes (TILs) (Tan et al.,) (14). However, in patients with early TNBC, PDL1 cannot predict the efficacy of ICI plus chemotherapy. Advanced TNBC patients with TMB≥10 mutations/Mb can achieve clinical benefits from pembrolizumab-based ICIs. Higher levels of TILs (e.g., ≥5% in the stroma) have been shown to predict a better response to pembrolizumab-based ICIs in TNBC. In this Research Topic, Wang et al. demonstrated that Ki67, in combination with the TIL level, can be used as a biomarker to predict the outcomes of TNBC patients with residual disease after neoadjuvant chemotherapy. They found that in TNBC patients with residual disease, TIL levels were correlated with favorable survival outcomes in patients with no change in Ki67, but not in patients with decreased Ki67 (Wang et al.). Even so, PDL1 remains the best, though imperfect, predictive biomarker for ICI efficacy (Tan et al.). Other biomarkers with predictive values, such as plasma IL-8 levels (15) and signatures generated from platelet-derived genes (16), are also worth exploring.

## Perspectives

Owing to the unique biological features and the aggressive clinical behavior of TNBC, more robust therapeutic approaches are urgently needed to improve patient outcomes. With the emergence of novel targeted therapeutic strategies, we are now seeing some improvements in clinical outcomes in TNBC. Unfortunately, the benefit of these novel therapies on the majority of TNBC patients remains subtle. To achieve improved outcomes, several issues need to be tackled with due attention. First, overcoming the limitations of immunotherapy through combination with other therapies such as cyclophosphamide, apatinib (inhibitor of VEGFR2), PARP inhibitors, oncolytic viruses, adoptive cell therapy, etc (17). Second, more personalized therapy should be implemented based on individual sequencing/multiomics profiling and/or drug efficacy test results (18). With more in-depth understanding of the molecular details behind the pathogenesis of TNBC and the utilization of the state-of-the-art technology, TNBC patients will expect better clinical outcomes in the future, hopefully not far from now.

## Author contributions

XD and CZ: Reviewing the literature and writing the original manuscript. FT and TR: Revising and editing the manuscript. Z-MS: Revising and reviewing the manuscript. All authors have read and approved the final submitted manuscript.

## Funding

This work was supported by the National Natural Science Foundation of China (82173374, 81872167) and Natural Science Foundation of Hunan (2019JJ40193, 2020JJ5386).

## Conflict of interest

The authors declare that the research was conducted in the absence of any commercial or financial relationships that could be construed as a potential conflict of interest.

## Publisher’s note

All claims expressed in this article are solely those of the authors and do not necessarily represent those of their affiliated organizations, or those of the publisher, the editors and the reviewers. Any product that may be evaluated in this article, or claim that may be made by its manufacturer, is not guaranteed or endorsed by the publisher.
